# Evidence of an ancient connectivity and biogeodispersal of a bitterling species, *Rhodeus notatus*, across the Korean Peninsula

**DOI:** 10.1038/s41598-020-57625-3

**Published:** 2020-01-23

**Authors:** Hari Won, Hyung-Bae Jeon, Ho Young Suk

**Affiliations:** 1 Department of Life Sciences, Yeungnam University, Gyeongsan, Gyeongsanbuk-do 38541 South Korea; 2 Department of Biology, Concordia University, 7141 Sherbrooke W., Montreal, Quebec H4B 1R6 Canada

**Keywords:** Evolutionary genetics, Ichthyology

## Abstract

The modern-day distribution of freshwater fishes throughout multiple rivers is likely the result of past migration during times when currently separate drainages were once connected. Here, we used mitochondrial and microsatellite analyses for 248 individuals of *Rhodeus notatus* collected from seven different rivers to obtain better understand historical gene flow of freshwater fish on the Korean Peninsula. Based on our phylogenetic analyses, this Korean species originated through the paleo-Yellow River from China and first colonized near the west coast. These genetic data also provided evidence of estuary coalescences among the rivers flowing to the west and southwest coast on well-developed continental shelf. In addition, the pattern of population structure revealed the biogeodispersal route from the west coast to the south coast. It could be inferred that massive migration was not involved in the formation of southern populations, since the signature of historical genetic drift was clearly observed. Our study is the first genetic attempt to confirm hypotheses describing the migration of freshwater species towards the end of East Asia, which have previously been developed using only geological reasoning.

## Introduction

Primary freshwater fish species living in separate river systems are not able to come into contact naturally. Even within a single river system, populations can be isolated by landscape structures^1–3^. The high degree of interpopulation genetic differentiation normally found in freshwater fish species has often been attributed to the fragmented nature of freshwater environments^4–7^. However, the distribution of freshwater fish species throughout multiple drainage basins is likely the result of past migration during times when currently separate drainages were once connected^8^. Insight into historical changes in past drainage systems and gene flow in a given region can be obtained by characterizing the pattern of genetic diversity and structure among populations that are currently spatially separated^9,10^.

The geography of the Korean Peninsula provides an excellent opportunity to explore the connections among the historical formation of freshwater systems and the colonization of freshwater fish^9,11–13^. This peninsula is a small but complex mountainous terrain that was created in the course of large-scale tectonic events from the early Triassic to early Miocene eras^14^. The sea between the western part of the Korean Peninsula and China is known to have been a lake into which the Yellow River once flowed; this lake was connected to the estuaries of many rivers flowing to the west coast on the Korean Peninsula^15^. Previous studies^11,15,16^ have claimed that many fish species found in these western-flowing rivers on the Korean Peninsula originated from the paleo-Yellow River system. Since the estuaries of these western-flowing rivers are connected or located in proximity on the huge continental shelf that is present around the west coast, it is presumed that freshwater fish species could move to other rivers whenever the sea levels became much lower than at present during the Pleistocene^17,18^. These rivers may have been isolated from the rivers that flow to the south coast where the continental shelf is not as well developed^9^. The coastal rivers on the east of the Korean Peninsula, which are surrounded by mountain ranges and are thought to have historically been influenced by the Amur River system from the north (east Russia), can also be regarded as an isolated region^13,15,19^. In this way, the freshwater system on the Korean Peninsula can be seen as having three independent historical subdistricts^15^.

*Rhodeus notatus* is a small freshwater bitterling species (Acheilognathidae; Cyprinoidea) native to China and the Korean Peninsula^15,20^. Before Kim revised the taxonomy of *Rhodeus*^15^, *R. notatus* of the Korean Peninsula was called *R. atremius* or *R. suigensis* in Japan or treated as a subspecies of *R. atremius* (i.e., *R. a. atremius* and *R. a. suigensis*)^21^. In fact, *R. notatus* has often been recognized as a member of the species complex including these two Japanese (sub)species and *R. fangi* in China, because these species share both the same number of chromosomes (i.e., 2n = 46), which is one pair less than other congeners, and other morphological features^22–24^. The Korean species was eventually synonymized with *R. notatus* after it was demonstrated that it had more morphological traits in common with Chinese *R. notatus* than other species within this species complex^15^. On the Korean Peninsula, *R. notatus* inhabits rivers flowing into the west and south coasts^25^. The distribution of this species preserves the historical imprints of migration through the paleo-Yellow River and the process of spreading to the freshwater system on the Korean Peninsula. The species could have migrated between rivers by sharing estuaries on the west coastal continental shelf^11^. Given that the continental shelf is not well-developed around the south coastal region, however, estuary coalescence might not be a major way to colonize the rivers flowing into the south coast, but instead it is more likely that gene flow occurred through the sharing of spatially proximal watersheds due to topographical erosion^13^. If so, large-scale population migration could not have been possible.

**Figure 1:**
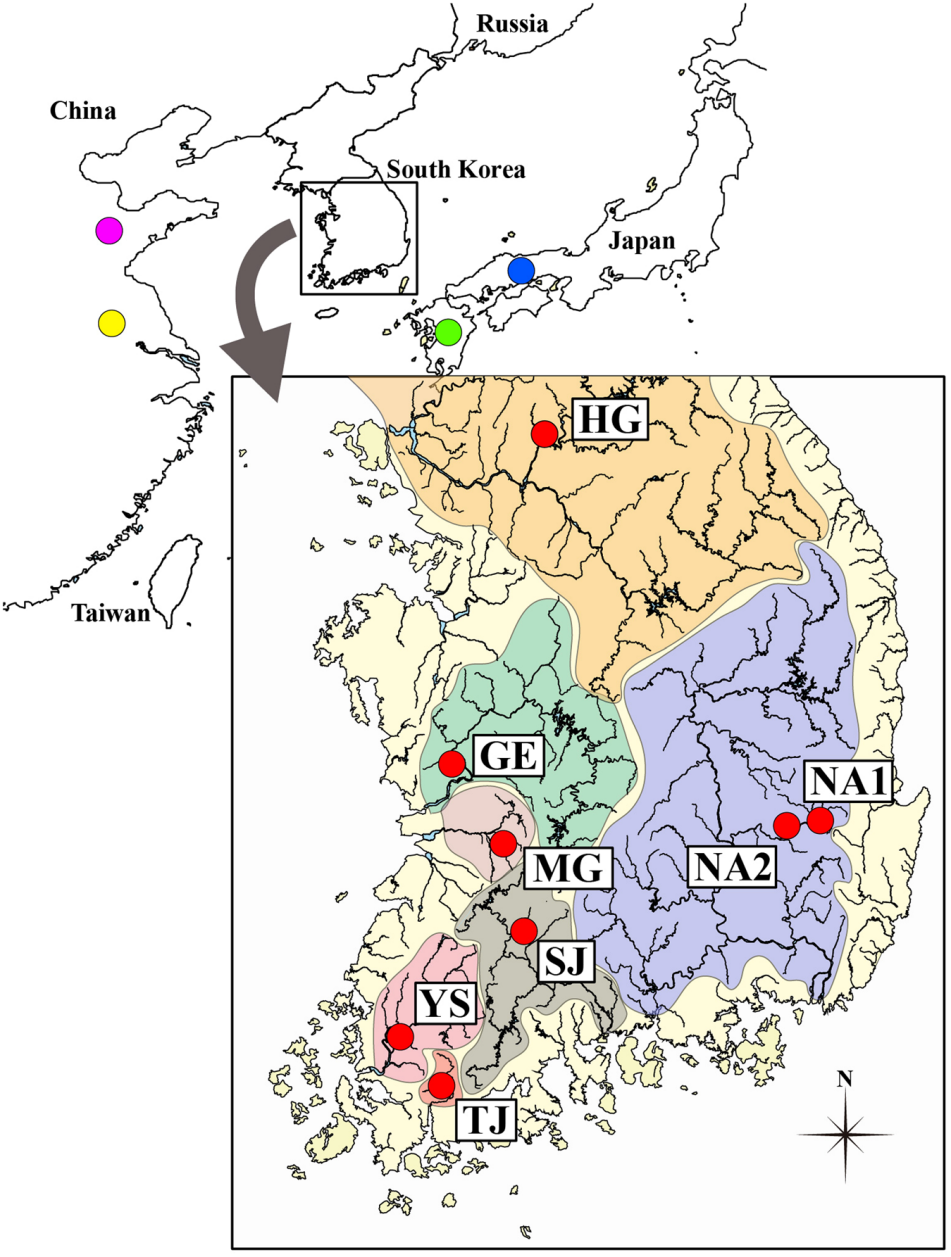
The collection sites of eight *Rhodeus notatus* populations from the Korean Peninsula. The locations of the reference species (pink: Chinese *R. notatus*; yellow: *R. fangi*; green; *R. atremius*; and blue: *R. suigensis*) used for comparison were also indicated on the East Asia map. The drainages on the peninsula were denoted by different colors. *Rhodeus notatus* populations comprise, from the west coastal region (western population group): HG, GE, MG; and from the south coastal region (southern population group): YS, TJ, SJ, NA1 and NA2. Geographic information of sampling localities is detailed in Table 1. The map was generated by Adobe Illustrator CC 2015 using a GIS shape file retrieved from inland water database in DIVA GIS (http://www.diva-gis.org/) and modified in QGIS v 2.16.3 in accordance with the guidelines suggested in the websites.

In this study, six mitochondrial and eight microsatellite loci were analyzed to estimate the level of intrapopulation genetic diversity, genetic structure among all major populations found on the Korean Peninsula and phylogenetic placement of *R. notatus* (Fig. 1). The results of these analyses were used to test four specific hypotheses about the geological and biogeographical history of freshwater ecosystem formation on the Korean Peninsula: i) *R. notatus* on the Korean Peninsula has originated from the paleo-Yellow River system, ii) the populations located in the rivers flowing into the west coast have genetic and phylogenetic signatures of estuary coalescence, iii) the populations of rivers flowing into the south coast where the continental shelf is not well-developed have signatures of history isolated from the western populations, and iv) the populations of the rivers flowing to the south coast, which probably formed through small-scale migration, have signatures of historical genetic drift.

## Results

### Mitochondrial analysis

Our mitochondrial analysis revealed low to moderate levels of genetic variation within and among populations of *R. notatus*. A total of 9, 15, 12, 8, 10 and 15 unique haplotypes were identified from the sequence alignments of COI (KY628232 – KY628240), NADH1 (KY628271 – KY628285), NADH2 (KY628259 – KY628270), 16 *S* (KY628241 – KY628248), 12 *S* (KY628249 – KY628258) and cyt *b* (KY628286 – KY628300), respectively (Supplementary Table S1). All populations except Seomjin (SJ), Nakdong (NA) 1 and NA2 contained their own unique haplotypes in all six mitochondrial loci (Supplementary Table S1). A total of 28 haplotypes were detected when the sequences of six mitochondrial loci were concatenated (Supplementary Table S2). Genetic diversity, estimated from concatenated sequences, varied considerably across populations, with haplotype number (hn) ranging from two in Youngsan (YS) and NA1 to six in Han (HG) and Tamjin (TJ); further, haplotype diversity (*h*) varied between 0.200 (±0.154; YS) and 0.844 (±0.103; HG and TJ; Table 1).

**Table 1:** Comparison of the genetic variability and historical demographic factors analyzed using six mitochondrial loci among populations and between two groups, western (HG, GE and MG) and southern genetic cluster (YS, SJ, ND1 and ND2) of *Rhodeus notatus* on the Korean Peninsula.

ID	River		*N*	hn	*h* (SD)	π (SD)	Tajima’s *D*	Fu’s *F*s
***Population***								
HG	Han	N37°75′57.45″/ E127°41′35.85″	10	6	0.844 (0.103)	0.00070 (0.00010)	1.166	−0.695
GE	Geum	N36°06′17.16″/ E126°45′50.55″	10	3	0.378 (0.181)	0.00009 (0.00005)	−1.401	−1.164
MG	Mangyoung	N35°51′15.33″/ E127°10′27.78″	10	4	0.644 (0.152)	0.00029 (0.00009)	−1.035	−0.312
YS	Youngsan	N35°00′34.83″/ E126°56′26.93″	10	2	0.200 (0.154)	0.00009 (0.00007)	−1.409	0.586
TJ	Tamjin	N34°40′21.43″/ E126°52′34.00″	10	6	0.844 (0.103)	0.00282 (0.00066)	1.393	2.839
SJ	Seomjin	N35°33′32.16″/ E127°21′52.34″	10	3	0.378 (0.181)	0.00009 (0.00005)	−1.401	−1.164
NA1	Nakdong	N35°55′01.14″/ E129°00′33.11″	10	2	0.467 (0.132)	0.00010 (0.00003)	1.050	0.818
NA2	Nakdong	N35°54′43.15″/ E128°50′13.64″	10	4	0.711 (0.117)	0.00804 (0.00143)	2.236*	12.319**
***Group***								
Western population group			30	13	0.883 (0.038)	0.0492 (0.00032)	2.061*	5.732*
Southern genetic cluster			36	9	0.795 (0.043)	0.00399 (0.00110)	−0.303	13.152*

The data comprise number of individuals, number of haplotypes, haplotype diversity (h with standard deviation), nucleotide diversity (π with standard deviation), Tajima’s D and Fu’s Fs. *P < 0.05.

**Figure 2:**
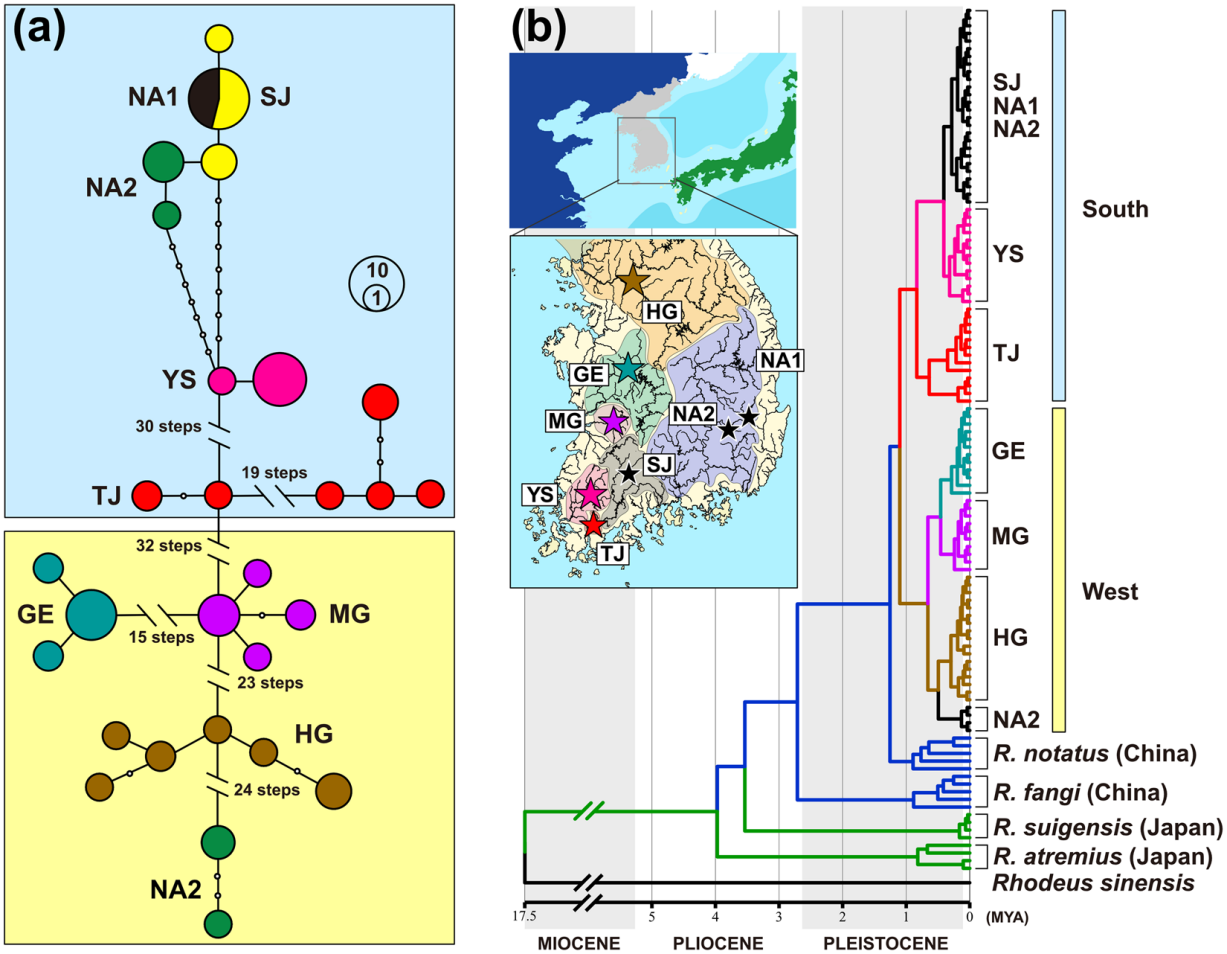
(**a**) The unrooted haplotype network generated based on the concatenated sequences of six mitochondrial loci used. (**b**) Time-calibrated Bayesian tree reconstructed by BEAST 2.3.2 using cyt *b* sequences of *R. notatus* and various acheilognathid species. This tree is an excerpt from the original tree made with all reference species (Supplementary Fig. S2 and Table S5). The 95% highest posterior confidence intervals of divergence times were estimated on all major nodes with blue bar. The biogeographic history of *R. notatus* and the closely related species was inferred under the dispersal-extinction-cladogenesis (DEC) model of geographic range evolution implemented in RASP 3.2. The colors of the nodes represent the most likely ancestral areas, which are the same color as the nine regions shown on the map.

We produced a haplotype network that illustrates genetic structure among the *R. notatus* populations we studied (Fig. 2a; Supplementary Fig. S1). All six mitochondrial loci data showed that haplotypes of populations SJ, NA1 and NA2 formed a cluster, around which population YS was closely located (Fig. 2a; Supplementary Fig. S1). Populations HG, Geum (GE) and Mangyoung (MG; western population group) were also closely related together despite the presence of strong genetic differentiation between them (Fig. 2a). In all loci except 12 *S*, population NA2 contained haplotypes that were distinct from the cluster of SJ, NA1 and NA2 and placed close to the haplotypes of western population group (Supplementary Fig. S1). Although population TJ geographically belongs to the southern population group, its haplotypes were quite distinct from those in other populations within this group (Supplementary Fig. S1).

In our phylogenetic trees reconstructed using six mitochondrial loci, all Korean *R. notatus* haplotypes were monophyletic (Supplementary Fig. S2). Among the Korean populations of *R. notatus*, western population group, populations HG, GE and MG, and populations SJ, NA1 and NA2 independently formed monophyletic clades (Supplementary Fig. S2). Populations TJ and YS clustered with populations SJ, NA1 and NA2 (Supplementary Fig. S2). When using only cyt *b*, the western population group and clades of SJ-NA1-NA2 were clearly revealed, and populations TJ and YS clustered with SJ-NA1-NA2 (Supplementary Fig. S3), suggesting that the populations TJ and YS were highly differentiated from these two clades as predicted from our haplotype network analysis (Fig. 2a; Supplementary Fig. S1).

In the BEAST^26^ tree reconstructed using cyt *b* data, all Korean *R. notatus* haplotypes were divided into western (HG, GE and MG) and southern population groups (TJ, YS, SJ, NA1 and NA2; Fig. 2b; Supplementary Fig. S3). Like the results of haplotype network analysis, some haplotypes of population NA2 were clustered together with the haplotypes of western population group (Fig. 2b). Korean *R. notatus* was resolved as the most likely sister taxon of Chinese *R. notatus* (Fig. 2b; Supplementary Fig. S3). The haplotypes of *R. fangi* were recovered as a sister group of Chinese and Korean *R. notatus*, with Japanese *R. atremius* and *R. suigensis* being placed at the ancestral position in the species complex (Fig. 2b; Supplementary Fig. S3). The root node for *R. notatus* species complex was estimated to be about 4 MYA (Fig. 2b; Supplementary Fig S3). The results of our BEAST tree and dispersal-extinction-cladogenesis (DEC) model analyses showed that *R. notatus* populations on the Korean Peninsula originated from China at about 1.25 MYA (Fig. 2b; Supplementary Fig S3). These analyses could also be used to reconstruct the historical processes of gradual dispersion and population formation. Right after the colonization on the west coast (population HG), *R. notatus* individuals likely migrated to the place where population TJ was located, at about 1.1 MYA (Fig. 2b; Supplementary Fig. S3). The individuals of population HG and population TJ appear to have migrated to form the populations of western and southern population groups, respectively (Fig. 2b; Supplementary Fig. S3).

**Figure 3:**
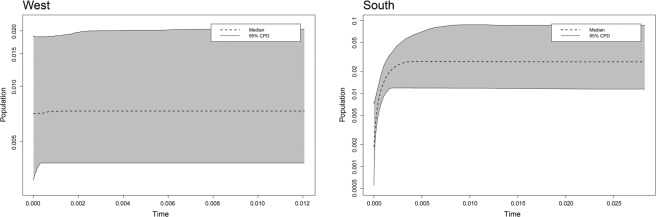
The results of extended Bayesian skyline plot analysis performed based on the concatenated sequences of six mitochondrial loci for western population group (HG, GE and MG; **a**) and southern genetic cluster (YS, SJ, NA1 and NA2; **b**) of *Rhodeus notatus*.

Mismatch distribution analysis (Supplementary Fig. S4) and extended Bayesian skyline plot (EBSP; Fig. 3) were performed for the two genetic clusters, western population group and the cluster of YS, SJ, NA1 and NA2 (southern genetic cluster; NA2 haplotypes clustered with western population group were not included) that were revealed in our haplotype network and BEAST tree analyses. The existence of strong genetic structure within western population group and southern genetic cluster likely produced multiple separate peaks in the mismatch distribution analysis (Supplementary Fig. S4). As expected from multiple peaks in the mismatch distribution graphs, both western population group and southern genetic clusters showed strong positive values of Tajima’s *D* and Fu’s *F*s (Table 1). In our EBSP, the effective population size greatly decreased around 100,000 years ago in southern genetic cluster, whereas no visible change was observed in western population group (Fig. 3). Except population NA2, no population showed significant values of Tajima’s *D* and Fu’s *F*s (Table 1). Significant positive values shown in population NA2 appear to be due to the existence of two different groups of haplotypes (Table 1; Supplementary Table S1).

### Microsatellite analysis

Microsatellite loci exhibited extensively different levels of diversity. For example, *Ak424* and *Ak462* showed considerably high genetic diversity with 22 and 25 alleles, respectively, but only two alleles were observed in *RC625* (Supplementary Table S3). As shown by the low heterozygosity of *Ak424*, the level of heterozygosity was not completely correlated with the allelic diversity (Supplementary Table S3). No significant signature of genotypic linkage disequilibrium was revealed from Fisher’s exact tests, indicating no possibility of physical linkage between loci or substructuring within populations. With the exception of *Ak424*, *F*_IS_ values did not deviate significantly from zero (Supplementary Table S3). However, *Ak424* was included in our further analyses, because such a result was thought to be due to the influence of the significant heterozygote deficiency in only a few populations (e.g., NA1) caused by the presence of null alleles, according to the results of Micro-Checker^27^. The overall level of interpopulation genetic differentiation was substantial with global *F*_ST_ = 0.361 and *R*_ST_ = 0.894 (see also Supplementary Table S3). The *R*_ST_ values were significantly higher than the mean permuted *R*_ST_ (and *F*_ST_) values with a few exceptions (Supplementary Table S3), indicating a significant contribution of stepwise mutations to the interpopulation genetic differentiation.

**Figure 4:**
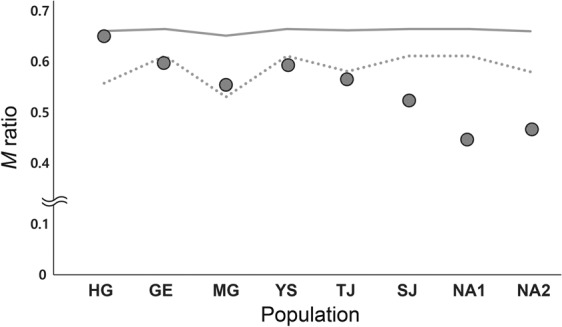
*M* ratio calculated from the microsatellite genotyping. Each circle indicates the average *M* ratio of each population of *Rhodeus notatus*. The solid and dot lines indicate the *M*c threshold calculated from parameter *θ* = 1 and *θ* = 10, respectively.

The genetic diversity of *R. notatus* varied across populations. A considerably higher level of genetic diversity was observed in populations TJ and YS, whereas population HG showed extremely lower diversity estimates than others (Table 2). At the population level, no significant heterozygote deficiency was observed (Table 2). In our maximum likelihood estimation of relatedness among individuals within populations using ML-Relate^28^, a lower level of family relationship was found in populations MG and TJ than others (Table 2). No signature of genetic bottleneck was found, with the exception of population NA1 that exhibited the signature of genetic bottleneck upon Wilcoxon’s heterozygosity excess test (Table 2). *M* ratio values varied between 0.446 and 0.650 (Fig. 4). Three populations in southern population group, SJ, NA1 and NA2, showed clearly lower values than the *M*_c_ values estimated for each population (Fig. 4).

**Table 2:** List of eight *Rhodeus notatus* populations on the Korean Peninsula and the microsatellite diversity estimates.

Pop	Drainage	*N*	*A*	*A*_R_	*H*_O_	*H*_E_	*F*_IS_	Freq Fam	Bottleneck	
									*P*	Mode
HG	Han	20	2.125	2.062	0.325	0.392	0.175	0.353	0.906	L-shaped
GE	Geum	40	4.125	3.229	0.342	0.377	0.094	0.254	0.990	L-shaped
MG	Mangyoung	15	4.875	4.758	0.429	0.539	0.211	0.124	0.844	L-shaped
YS	Youngsan	40	4.250	3.596	0.588	0.613	0.042	0.255	0.469	L-shaped
TJ	Tamjin	27	7.125	5.963	0.576	0.617	0.066	0.148	0.273	L-shaped
SJ	Seomjin	40	3.125	2.535	0.421	0.460	0.085	0.291	0.469	L-shaped
NA1	Nakdong	40	2.750	2.559	0.405	0.505	0.200	0.282	0.004*	L-shaped
NA2	Nakdong	26	3.625	3.396	0.509	0.531	0.043	0.268	0.010	L-shaped

Data include population ID (Pop), drainage basin (Drainage), total number of individuals analyzed (*N*), total number of alleles per locus (*A*), allelic richness (*A*_R_), observed (*H*_O_) and expected (*H*_E_) heterozygosities, fixation indices (*F*_IS_), the frequency of family relationship (obtained using ML-Relate) and the signature of genetic bottleneck (obtained using Bottleneck; *P*: Wilcoxon sign-rank test under the TPM; Mode: mode-shift deviation from the typical L-shaped distribution of allelic frequencies).

*P < 0.00625.

**Figure 5:**
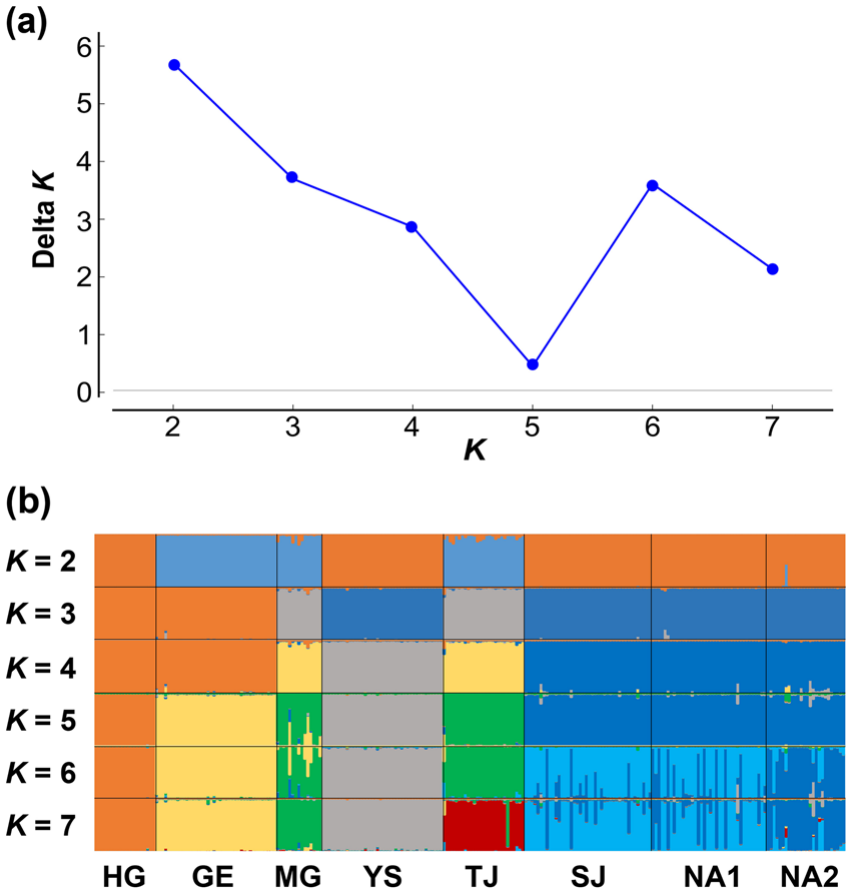
Population structure of *Rhodeus notatus* estimated from microsatellite genotyping. (**a**) The delta *K* method indicated that the most reliable number of clusters was two or six. (**b**) The population structure estimated in Structure indicated the existence of variable numbers of genetic clusters (*K* = 2 to 7).

The overall pairwise *F*_ST_ and *R*_ST_ values among populations were substantially high with some exceptions (Table 3), as predicted from the great global *F*_ST_ and *R*_ST_ values (Supplementary Table S3). Populations HG and GE in western population group showed extremely high *F*_ST_ and *R*_ST_ values in all pairwise comparisons (Table 3). Populations SJ, NA1 and NA2 exhibited a quite low genetic differentiation from each other, and populations TJ and YS seemed to have relatively higher genetic affinity to those populations (Table 3). Overall, multilocus *R*_ST_ values were significantly higher than *F*_ST_ and mean permuted *R*_ST_ values (*R*_ST_ = 0.894, p*R*_ST_ = 0.598; *P* = 0.006), suggesting the significant contribution of stepwise mutations to interpopulation genetic differentiation at the microsatellite loci. However, the average pairwise *R*_ST_ values were much lower than those of pairwise *F*_ST_ in the comparisons among populations of southern population group (Table 3). It was difficult to determine the optimal number of genetically distinguishable groups for our Bayesian Structure analysis (Fig. 5a). Most populations seem to have distinctly different genetic characteristics, although populations SJ, NA1 and NA2 tended to be closely tied together (Fig. 5b). Populations MG and TJ also showed relatively high genetic affinity with each other in this analysis (Fig. 5b). Upon PCA analysis, populations were likely subdivided into three groups (HG, GE-MG and the remainder), though a slight overlap occurred due to the genetic affinity between populations MG and TJ (Supplementary Fig. S5).

**Table 3:** Summary of pairwise microsatellite genetic differentiation of eight *Rhodeus notatus* populations from the Korean Peninsula.

	HG	GE	MG	YS	TJ	SJ	NA1	NA2
HG		0.549	0.535	0.527	0.519	0.555	0.485	0.497
GE	0.915		0.247	0.435	0.246	0.378	0.321	0.310
MG	0.785	0.762		0.322	0.181	0.265	0.212	0.155
YS	0.778	0.674	0.365		0.277	0.222	0.229	0.241
TJ	0.827	0.698	0.222	0.141		0.178	0.147	0.131
SJ	0.878	0.804	0.374	0.105	0.083		0.056	0.140
NA1	0.888	0.796	0.395	0.136	0.068	0.019^NS^		0.079
NA2	0.843	0.688	0.241	0.161	0.011^NS^	0.180	0.166	

Estimates of *F*_ST_ appear above the diagonal and estimates of *R*_ST_ appear below the diagonal.

Note: All comparisons are significantly different from zero (*P* < 0.05) except those denoted by ‘NS’.

**Figure 6:**
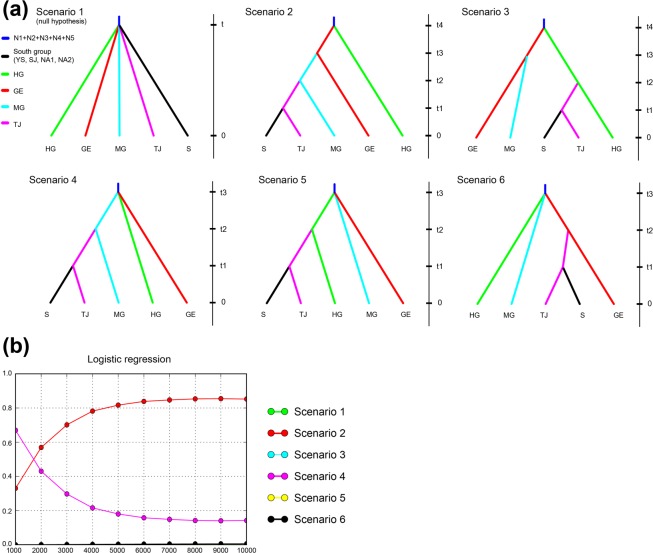
The results of Approximate Bayesian Computation (ABC) analysis implemented in DIYABC to identify the origin of population TJ from western population group. (**a**) Six (including null) biogeographic scenarios were designed and tested in DIYABC analysis. (**b**) The most likely scenario with the highest posterior probability was identified by performing a weighted logistic regression of each scenario probability to compare the deviations between simulated and observed summary statistics. The most likely was scenario 2, followed scenario 4, while possibilities for other scenarios were negligible.

An Approximate Bayesian Computation (ABC) approach was applied to identify historical pathway from western population group to population TJ using DIYABC^29^. Five possible scenarios were designed and tested with logistic regressions of simulated against observed data (Fig. 6). These five scenarios represent three different hypotheses about the origin of population TJ; the origin in scenarios 2 and 4, scenarios 3 and 5, and scenario 6 were MG, HG and GE, respectively (Fig. 6a). Our DIYABC analysis revealed that the most likely was scenario 2, followed scenario 4, while possibilities for other scenarios were negligible (Fig. 6), indicating that population TJ or the southern population group split from an ancestor of MG. These results were correlated with the overall pattern of pairwise *F*_ST_ and *R*_ST_ values (Table 3; Supplementary Fig. S5).

## Discussion

The average intrapopulation genetic diversity of the eight *Rhodeus notatus* populations examined in this study is quite low, given that fewer than two haplotypes per mitochondrial locus were observed in a population. Although no direct comparison was attempted using the same loci, the average number of microsatellite alleles per locus in a population was much below the known freshwater fish average^21,30–32^. *Tanakia somjinensis*, also a member of Acheilognathidae, has a much higher microsatellite diversity than *R. notatus*^29^. Given that *T. somjinensis* is a highly restricted Korean endemic species and is protected by Korean authority^33^, careful monitoring and management strategies for *R. notatus* will likely be required.

The results of our phylogenetic analysis revealed that two Japanese species, *R. suigensis* and *R. atremius* were the earliest branches from the clade of *R. notatus* species complex, suggesting that this complex originally evolved near Japan, or that peripheral speciation occurred around Japan from a widely distributed common ancestor. This prediction aligns with the previous claims that there have been widespread migrations of freshwater species between China and Japan via the Korean Peninsula^34–36^. In addition, the clear phylogenetic division of the Japanese species from others may be related to the geological process in which Japanese Archipelago has become completely separated from the continent by the sea. It can be assumed from our data that *R. notatus* on the Korean Peninsula originated through the paleo-Yellow River, based on the results of historical distribution estimation using our DEC model analysis and its phylogenetic affinity to the Chinese populations of *R. notatus* and *R. fangi*.

Given that Chinese *R. notatus* haplotypes are distinct from all Korean *R. notatus* haplotypes, it is difficult to determine the origin of *R. notatus* on the Korean Peninsula using only the degree of genetic affinity to Chinese haplotypes. The presence of strong genetic structure in the western population group demonstrates that there has been little active gene flow among the populations formed by paleo-yellow confluence. From the results of our haplotype network, DEC model and DIYABC analyses, we attempted to reconstruct the historical pathway to form southern population group from western population group. Solely based on our DEC model results, some individuals from population HG further migrated south to form a peri-Tamjin River population (TJ). It might be possible that populations GE and MG might have formed from the dispersal from population HG; both our haplotype network and microsatellite analyses indicate that the southern population group and population TJ had the closest genetic affinity with population MG. These results were also supported by DIYABC simulation and showed, at least indirectly, the historical trace of a dispersal over time from north to south on the west coastal continental shelf and to southern coastal rivers through population TJ.

The genetic diversity of population TJ was high compared to other populations in both mitochondrial and microsatellite loci. Although the mitochondrial diversity of population HG was also high, its microsatellite diversity was substantially lower than others, with half of the loci being completely fixed to a single allele. Models of neutral evolution suggest that genetic diversity is roughly proportional to the size of a population^37,38^; however, our data do not suggest that population TJ has even been particularly large throughout its history. This suggests that the region encompassing population TJ might have received migrants from several western coastal populations on the Korean Peninsula. Two slightly differentiated haplogroups were present in the monophyletic population TJ can be considered as evidence of gene flow from multiple isolated regions. Although the Korean Peninsula has never been covered with glaciers during Pleistocene glacial advances, cold and arid weather had persisted long even in the central region^39^ where the populations HG and GE are located. Under these historical circumstances, there may have been massive migration of freshwater species to more favorable regions, and large populations may have been formed in the southwestern corner.

Although population YS is also located on the southwestern corner of the Korean Peninsula, no clear signature of historical gene flow was identified from the western population group. Our genetic analyses revealed that population YS showed some degree of genetic affinity with population TJ and with the southern populations, SJ, NA1 and NA2. This result suggested the presence of a biogeographic pathway from population TJ to YS and then to the southern populations. The biogeographic route of freshwater systems along the south coast has never been reported to date, and in this respect, our results are of great importance.

As noted above, unlike the west coast of the Korean Peninsula, the continental shelf is not well developed around the south coast, and opportunities for past rivers to share estuaries with sea level fluctuation may not have existed. Indeed, studies have reported on the signature of gene flow by watershed capture between southern-flowing rivers^25,40^. Massive gene flow might not have been possible through watershed capture, as predicted in the introduction. The results of our various analyses help prove this assumption. First, our *M*-ratio data showed the signature of historical bottlenecks in populations SJ, NA1 and NA2. Second, at a regional level, the relative contribution of stepwise mutations to genetic differentiation among populations of southern population groups was much less pronounced; it is more likely that genetic drift was the cause of the differentiation among these populations. Finally, as evidenced by our EBSP, the populations of southern population group had likely undergone historical size decline.

Population NA2 contained rather unusual haplotypes that were genetically close to those of the western population group, especially population HG. It is not reasonable to suspect that such a genetic composition was caused by anthropogenic introduction of this species living in the Han River into the Nakdong River, because these two populations did not share the identical haplotypes. In addition, such a genetic pattern was not observed in our microsatellite analysis. Considering that the watershed locations of the Han and Nakdong Rivers are spatially proximal, it is possible that these haplotypes of population NA2 were naturally distributed; however, no direct evidence has yet been reported in support of this hypothesis.

We were able to address our major questions regarding the geographic origins of extant diversity among populations of *R. notatus*. First, our study revealed that *R. notatus* on the Korean Peninsula originated through the paleo-Yellow River system from China. Second, our genetic data demonstrated that the rivers flowing into the west and southwest coasts had formed confluence with the paleo-Yellow River system. However, the presence of a strong and complicated genetic structure within the western population group implies that historical gene flow among populations was not highly active, probably because long-term perfect confluence has not been formed, and the topographical structure on the continental shelf has strongly influenced on the pattern of gene flows. Third, the rivers flowing to the south coast seem to have been completely isolated from the confluence on the west and southwest, considering the great genetic difference between the western and southern population groups. Our data demonstrate that populations on the southwest corner had served as a biogeodispersal passage from west to south on the peninsula. Finally, migration among the populations of southern population group might have been possible only by small-scaled watershed captures, which was supported by the signature of historical genetic drift found in populations SJ, NA1 and NA2 from our results. This study represents the first use of molecular genetics to characterize the process of shaping contemporary distribution of freshwater species in the southwestern part of the Korean Peninsula. The inferences about the various fine-scale biogeographic pathways obtained here can be verified by a comparative study in the future with taxa having similar geographical distribution, for example, *R. sinensis* of the same genus.

## Materials and Methods

### Sampling and extraction

*Rhodeus notatus* individuals used in our genetic studies were 248 specimens stored at the Department of Life Sciences, Yeungnam University. Those specimens were collected from eight populations on seven different river basins flowing into west coast (western population group: HG, GE and MG) and south coast (southern population group: TJ, YS, SJ, NA1 and NA2; Fig. 1) from 2009 to 2011 in accordance with the Inland Water Fisheries Act and Wildlife Protection and Management Act of the Republic of Korea. The entire procedure of this study was approved by the Yeungnam University Institutional Animal Care and Use Committee (Protocol # 2015013). Small fin-clips cut from caudal fin of all individuals were used as tissue samples for our genetic analysis. Genomic DNA was extracted using a Wizard Genomic DNA purification kit (Promega, Madison, WI, USA).

### Mitochondrial sequencing

Six mitochondrial loci, cytochrome oxidase subunit 1 (COI), cytochrome *b* (cyt *b*), NADH dehydrogenase subunit 1 (NADH1), NADH dehydrogenase subunit 2 (NADH2), 12 *S r*RNA (12 *S*) and 16 *S r*RNA (16 *S*), were sequenced for 10 selected *R. notatus* individuals per population. Information on the mitochondrial primer sets used in this study^41–45^ was provided in Supplementary Table S4. PCR was conducted using a 25 µl mixture consisting of 10–50 ng DNA, 1× *Taq* buffer (containing 2.5 mM MgCl_2_), 0.25 mM dNTPs, 1 μM of each primer and 0.25 units of *Taq* DNA polymerase (Solgent Inc, Daejeon, South Korea). Thermal cycling was composed of an initial denaturation at 95 °C for 5 min, 35 cycles of a denaturation at 95 °C for 30 sec, an annealing at 54 or 56 °C (see Supplementary Table S4) for 30 sec, at an extension at 72 °C for 45 sec, and an extra extension at 72 °C for 10 min. The PCR products were electrophoresed on 1.5% agarose gels to check the quality, purified using Wizard Genomic DNA purification kit (Promega, Madison, WI, USA) and sequenced by Genotech Inc (Daejeon, South Korea) on an ABI3730XL (Applied Biosystmes, Foster City, CA, USA) with BigDye Terminator 3.1 Cycle Sequencing Kit (Applied Biosystems).

### Mitochondrial analyses

All mitochondrial sequences were rechecked through BLAST searches and aligned using Geneious 9.1.8^46^. The coding loci, including COI, cyt *b*, NADH1 and NADH2, were examined against the inferred reading frame for the corresponding proteins using MEGA 6.06^47^. The intrapopulation genetic diversity was quantified by the number of haplotypes (hn), haplotype diversity (*h*)^48^ and nucleotide diversity (π)^48^ estimated using DnaSP 5.10^49^. The unrooted haplotype network was generated for the six mitochondrial loci and the concatenated sequences of all loci based on the connection limit above 0.95 in probability using TCS 1.263^50^ to analyze the distribution of haplotype diversity.

Phylogenetic trees were generated under two different algorithms, Bayesian inference (BI) and maximum likelihood (ML). All six mitochondrial loci were used in the reconstruction of phylogenetic trees. BI tree analysis was performed using MrBayes 3.2^51^, and GTR + I + G was chosen as the best-fit substitution model by jModelTest 2^52^ as the best-fitting nucleotide substitution model. BI analysis consisted of two parallel runs of 80 million Markov Chain Monte Carlo (MCMC) generations with sampling every 1,000 steps. The consensus tree for each data set was generated after removing the first 25% of sampled trees as burn-in. The Bayesian posterior probabilities (in percent) were presented as the node confidence in the tree. The ML tree analysis was conducted using IQ-Tree 1.3.10 with 10,000 ultrafast bootstrap replicates^53^. The best-fit substitution model was chosen to be GTR + I + G based on Bayesian Information Criterion (BIC) using ModelFinder^54^ implemented in IQ-Tree. Cyt *b* sequences were used in divergence time estimation using BEAST 2.3.0^26^. This mitochondrial locus was used for this analysis because the cyt *b* sequence was most commonly reported in bitterling and cyprinid species that could be used as comparative materials from China and Japan and outgroups; see Supplementary Table S5. For the BEAST analysis, two independent runs of MCMC were performed based on 100 million generations, with sampling every 1,000^th^ generations, using GTR + I + G selected by jModelTest 2 as the best-fitting nucleotide substitution model. A Yule tree prior and an uncorrelated lognormal relaxed-clock were implemented on this analysis. Calibration of the molecular clock was conducted by using the earliest fossil record data of Acheilognathidae^55,56^ for the basal node of this family (23.0 Mya; normal distribution). Tracer 1.657^57^ was used to examine the convergence of run parameters by determining whether ESSs (effective population sizes) exceeded 200. The maximum clade credibility tree with median heights was generated using TreeAnnotator 2.3.0^58^, after discarding the first 25% of trees as burn-in. FigTree 1.4.2^59^ was used to visualize the topology of the consensus tree generated with divergence times. The biogeographic history of *R. notatus* and the closely related species was explored under the dispersal-extinction-cladogenesis (DEC) model of geographic range evolution^60^ implemented in RASP 3.2^61^. The DEC model was used to assess the likelihood of all possible ancestral distributions at a given node^57^. A biogeographic model was constructed by coding each species or population used in the BEAST analysis as occurring in one of the nine regions including the seven drainage basins in which Korean *R. notatus* individuals were collected, China and Japan. The tests of DEC were performed based on the parameters obtained from our BEAST analysis, and the results were visualized on the tree using Affinity Designer 1.6.1 (Serif Ltd; https://affinity.serif.com/en-us/).

All six mitochondrial loci were used to find the best approximation regarding the historical demographic pattern for each *R. notatus* population and genetic cluster based on three different methods. First, Tajima’s *D*^62^ and Fu’s *F*s^63^ were calculated using DnaSP. Second, mismatch distribution analysis was carried out to show the observed distribution of pairwise differences between mitochondrial sequences under the model of population expansion^64^ using Arlequin 3.5^65^ for each genetic cluster. Finally, extended Bayesian skyline plot (EBSP) was created using BEAST for each genetic cluster^66^ to evaluate historical demographic changes of effective population size over historical time under the HKY model and strict-clock model with mutation rates (1% per million years)^67^. The EBSP analysis was performed for 10 million generations with sampling every 1,000 steps and discarding the first 25% as burn-in. Tracer was used to assess the convergence between runs by examining whether ESSs exceeded 200 as an indicator.

### Microsatellite genotyping

All samples were genotyped using eight previously reported microsatellite primer sets (Supplementary Table S3)^26,68,69^. The 5′ end of each forward primer was fluorescently labelled with either FAM, HEX or NED (Applied Biosystems Life Technologies, Carlsbad, CA, USA). PCR was conducted using a 10 µl mixture containing 1 µl of extracted DNA, 1× *Taq* buffer (containing 2.5 mM MgCl_2_), 0.25 mM dNTPs, 1 µM of each primer and 0.15 units of *Taq* DNA polymerase (Solgent). Thermal cycling consisted of an initial denaturation at 95 °C for 5 min, 35 cycles of a denaturation at 95 °C for 30 sec, an annealing at 54–58 °C for 30 sec, an extension at 72 °C for 45 sec, and an final extension at 72 °C for 10 min. The fluorescently labeled PCR products were genotyped on an ABI 3730xl Genetic Analyzer by Biomedic Inc (Bucheon, South Korea) and scored using GeneMapper 3.7 (Applied Biosystems). Allele size was determined using the Peak Scanner 1.0 (Applied Biosystems).

### Microsatellite analyses

Genetic diversity for each locus and population was quantified with the mean or total number of alleles per locus, allelic richness, observed and expected heterozygosities and fixation index (*F*_IS_) estimated in Arlequin, Fstat 2.9.3.2^70^ and Genepop 4.2^71^. Genepop was used to detect whether the genotype frequencies were deviated from Hardy-Weinberg equilibrium for each locus–population combination using the Fisher’s exact test based on Markov chain parameters with 1000 batches and 10,000 iterations per batch^72^. Fisher’s exact test of linkage disequilibrium between pairs of loci was performed with the Markov chain algorithm under the null hypothesis of independence using Genepop. Microsatellite genotypes were checked using Micro-Checker 2.2.3^27^.

The expected percentage of family (full-sib, half-sib and parent-offspring) relatedness for each population was determined using ML-Relate. A significant excess of expected heterozygosity under the mutation-drift equilibrium relative to expected heterozygosity under Hardy-Weinberg equilibrium was checked based on the Wilcoxon sign-rank test under the TPM (two phase model) with a setting of 70% stepwise mutations model (SMM) and 30% infinite alleles model (IAM) using Bottleneck v1.2^73^. In addition, Bottleneck was used to examine mode-shift deviation from the typical L-shaped distribution of allelic frequencies^74^. Both Bottleneck analyses were used to test whether populations may have recently undergone significant size reduction. The *M* ratio, which is the mean ratio of the number of alleles to the range in allele size^75^, and a critical value of *M* (*M*_c_) were quantified for each population using Arlequin to test whether populations have historically experienced a significant reduction in size^75^. *M*_c_ was calculated with default values and *θ* (=4*N*_e_μ) = 1 and 10 under a TPM constraining the model by defining 80% of mutations as conforming to a stepwise mutation model and 20% as an infinite allele model. Critical significance values in all statistics were corrected for multiple comparisons based on the Bonferroni procedure^76^.

Genetic distance among populations was quantified using global *F*_ST_^77^ and *R*_ST_^78^, as well as pairwise *F*_ST_^77^ and *R*_ST_^79^ estimated by Arlequin and Genepop. A randomization procedure of allele sizes was then conducted using SPAGeDi v1.1^80^, whereby the different allele sizes found at a locus were subjected to 2,000 random permutations to examine whether the allele sizes from stepwise mutation contributed to population differentiation^81^. Bayesian clustering analysis with the software Structure v2.3.4^82^ was used to estimate the degree of structuring among populations. Before analysis, the putative numbers of genetically distinguishable groups (*K*) were predicted by the delta *K* method^83^ implemented in Structure Harvester v0.6.94^84^. Ten independent MCMC runs were performed for each *K* with 4 × 10^5^ iterations after a burn-in of 10^5^ iterations. To visualize the relationship between individuals and populations based on the best linear combination of allele frequencies, principal component analysis (PCA) was implemented using GENETIX v4.05^85^. To test competing hypotheses about biogeographic pathways leading to population TJ from western population groups showing strong genetic structure, an Approximate Bayesian Computation (ABC) approach was taken, implemented in the software DIYABC v.2.1.0^29^. A total of five scenarios with a null hypothesis were tested (Fig. 6), and the factor to distinguish between scenarios was tree topology. A total of 1,000,000 simulated datasets were grouped according to each scenario. The probability of each scenario was measured by performing a weighted logistic regression of each scenario probability to compare the deviations between simulated and observed summary statistics to find the most likely scenario.

## Supplementary information

Supplementary Information.
