# Supplementary material for: Evidence of an ancient connectivity and biogeodispersal of a bitterling species, Rhodeus notatus, across the Korean Peninsula
Source: Sci Rep. 2020 Jan 23;10:1011. doi: 10.1038/s41598-020-57625-3 (PMC6978382; doi:10.1038/s41598-020-57625-3)
Supplement: Supplementary file 1 — Supplementary Information. [file 41598_2020_57625_MOESM1_ESM.docx]

**Evidence of an ancient connectivity and biogeodispersal of a bitterling species, *Rhodeus notatus*, across the Korean Peninsula**

Hari Won^1^, Hyung-Bae Jeon^1,2^, Ho Young Suk^1,*^

^1^ Department of Life Sciences, Yeungnam University, Gyeongsan, Gyeongsanbuk-do 38541, South Korea

^2^ Department of Biology, Concordia University, 7141 Sherbrooke W., Montreal, Quebec H4B 1R6, Canada

* Correspondence and requests for materials should be addressed to H. Y. Suk (email: [hsuk@ynu.ac.kr](mailto:hsuk@ynu.ac.kr))

Running Title: Genetic and phylogenetic structure of *Rhodeus notatus*

Submitted to *Scientific Reports*

December 12, 2019

Table S1. Distribution pattern of six mitochondrial loci haplotypes among populations of *Rhodeus notatus* on the Korean Peninsula; western and southern population groups were denoted by western and southern, respectively.

| Locus | Haplotype | Frequency | Western | | |  | Southern | | | | |
| --- | --- | --- | --- | --- | --- | --- | --- | --- | --- | --- | --- |
|  |  |  | HG | GE | MG |  | YG | TJ | SJ | NA1 | NA2 |
| COI | H1 | 10 | 10 |  |  |  |  |  |  |  |  |
|  | H2 | 10 |  | 10 |  |  |  |  |  |  |  |
|  | H3 | 8 |  |  | 8 |  |  |  |  |  |  |
|  | H4 | 2 |  |  | 2 |  |  |  |  |  |  |
|  | H5 | 11 |  |  |  |  | 10 |  |  |  | 1 |
|  | H6 | 6 |  |  |  |  |  | 6 |  |  |  |
|  | H7 | 4 |  |  |  |  |  | 4 |  |  |  |
|  | H8 | 25 |  |  |  |  |  |  | 10 | 10 | 5 |
|  | H9 | 4 |  |  |  |  |  |  |  |  | 4 |
| NADH1 | H1 | 5 | 5 |  |  |  |  |  |  |  |  |
|  | H2 | 5 | 5 |  |  |  |  |  |  |  |  |
|  | H3 | 9 |  | 9 |  |  |  |  |  |  |  |
|  | H4 | 1 |  | 1 |  |  |  |  |  |  |  |
|  | H5 | 8 |  |  | 8 |  |  |  |  |  |  |
|  | H6 | 2 |  |  | 2 |  |  |  |  |  |  |
|  | H7 | 10 |  |  |  |  | 10 |  |  |  |  |
|  | H8 | 4 |  |  |  |  |  | 4 |  |  |  |
|  | H9 | 2 |  |  |  |  |  | 2 |  |  |  |
|  | H10 | 2 |  |  |  |  |  | 2 |  |  |  |
|  | H11 | 1 |  |  |  |  |  | 1 |  |  |  |
|  | H12 | 1 |  |  |  |  |  | 1 |  |  |  |
|  | H13 | 26 |  |  |  |  |  |  | 10 | 9 | 6 |
|  | H14 | 3 |  |  |  |  |  |  |  |  | 3 |
|  | H15 | 1 |  |  |  |  |  |  |  |  | 1 |
| NADH2 | H1 | 5 | 5 |  |  |  |  |  |  |  |  |
|  | H2 | 5 | 5 |  |  |  |  |  |  |  |  |
|  | H3 | 10 |  | 10 |  |  |  |  |  |  |  |
|  | H4 | 9 |  |  | 9 |  |  |  |  |  |  |
|  | H5 | 1 |  |  | 1 |  |  |  |  |  |  |
|  | H6 | 9 |  |  |  |  | 9 |  |  |  |  |
|  | H7 | 1 |  |  |  |  | 1 |  |  |  |  |
|  | H8 | 7 |  |  |  |  |  | 7 |  |  |  |
|  | H9 | 3 |  |  |  |  |  | 3 |  |  |  |
|  | H10 | 26 |  |  |  |  |  |  | 10 | 10 | 6 |
|  | H11 | 3 |  |  |  |  |  |  |  |  | 3 |
|  | H12 | 1 |  |  |  |  |  |  |  |  | 1 |
| 16*S r*RNA | H2 | 10 | 10 |  |  |  |  |  |  |  |  |
|  | H3 | 10 |  | 10 |  |  |  |  |  |  |  |
|  | H4 | 10 |  |  | 10 |  |  |  |  |  |  |
|  | H5 | 10 |  |  |  |  | 10 |  |  |  |  |
|  | H6 | 7 |  |  |  |  |  | 7 |  |  |  |
|  | H8 | 3 |  |  |  |  |  | 3 |  |  |  |
|  | H1 | 26 |  |  |  |  |  |  | 10 | 10 | 6 |
|  | H7 | 4 |  |  |  |  |  |  |  |  | 4 |
| 12*S r*RNA | H1 | 5 | 5 |  |  |  |  |  |  |  |  |
|  | H2 | 5 | 5 |  |  |  |  |  |  |  |  |
|  | H3 | 10 |  | 10 |  |  |  |  |  |  |  |
|  | H4 | 14 |  |  | 10 |  |  |  |  |  | 4 |
|  | H5 | 10 |  |  |  |  | 10 |  |  |  |  |
|  | H6 | 4 |  |  |  |  |  | 4 |  |  |  |
|  | H7 | 3 |  |  |  |  |  | 3 |  |  |  |
|  | H8 | 3 |  |  |  |  |  | 3 |  |  |  |
|  | H9 | 20 |  |  |  |  |  |  | 10 | 10 |  |
|  | H10 | 6 |  |  |  |  |  |  |  |  | 6 |
| Cyt *b* | H1 | 6 | 6 |  |  |  |  |  |  |  |  |
|  | H2 | 3 | 3 |  |  |  |  |  |  |  |  |
|  | H3 | 1 | 1 |  |  |  |  |  |  |  |  |
|  | H4 | 9 |  | 9 |  |  |  |  |  |  |  |
|  | H5 | 1 |  | 1 |  |  |  |  |  |  |  |
|  | H6 | 9 |  |  | 9 |  |  |  |  |  |  |
|  | H7 | 1 |  |  | 1 |  |  |  |  |  |  |
|  | H8 | 10 |  |  |  |  | 10 |  |  |  |  |
|  | H9 | 6 |  |  |  |  |  | 6 |  |  |  |
|  | H10 | 3 |  |  |  |  |  | 3 |  |  |  |
|  | H11 | 1 |  |  |  |  |  | 1 |  |  |  |
|  | H12 | 1 |  |  |  |  |  |  | 1 |  |  |
|  | H13 | 15 |  |  |  |  |  |  | 8 | 7 |  |
|  | H14 | 10 |  |  |  |  |  |  | 1 | 3 | 6 |
|  | H15 | 4 |  |  |  |  |  |  |  |  | 4 |

Table S2. Distribution pattern of mitochondrial haplotypes obtained from the concatenated loci data among populations of *Rhodeus notatus* on the Korean Peninsula; western and southern population groups were denoted by western and southern, respectively.

| Haplotype | Frequency | Western | | | |  | Southern | | | | |
| --- | --- | --- | --- | --- | --- | --- | --- | --- | --- | --- | --- |
|  |  | HG | GE | | MG |  | YG | TJ | SJ | NA1 | NA2 |
| H1 | 4 | 4 | |  |  |  |  |  |  |  |  |
| H2 | 2 | 2 | |  |  |  |  |  |  |  |  |
| H3 | 1 | 1 | |  |  |  |  |  |  |  |  |
| H4 | 1 | 1 | |  |  |  |  |  |  |  |  |
| H5 | 1 | 1 | |  |  |  |  |  |  |  |  |
| H6 | 1 | 1 | |  |  |  |  |  |  |  |  |
| H7 | 8 |  | | 8 |  |  |  |  |  |  |  |
| H8 | 1 |  | | 1 |  |  |  |  |  |  |  |
| H9 | 1 |  | | 1 |  |  |  |  |  |  |  |
| H10 | 6 |  | |  | 6 |  |  |  |  |  |  |
| H11 | 2 |  | |  | 2 |  |  |  |  |  |  |
| H12 | 1 |  | |  | 1 |  |  |  |  |  |  |
| H13 | 1 |  | |  | 1 |  |  |  |  |  |  |
| H14 | 9 |  | |  |  |  | 9 |  |  |  |  |
| H15 | 1 |  | |  |  |  | 1 |  |  |  |  |
| H16 | 4 |  | |  |  |  |  | 4 |  |  |  |
| H17 | 2 |  | |  |  |  |  | 2 |  |  |  |
| H18 | 1 |  | |  |  |  |  | 1 |  |  |  |
| H19 | 1 |  | |  |  |  |  | 1 |  |  |  |
| H20 | 1 |  | |  |  |  |  | 1 |  |  |  |
| H21 | 1 |  | |  |  |  |  | 1 |  |  |  |
| H22 | 15 |  | |  |  |  |  |  | 8 | 7 |  |
| H23 | 4 |  | |  |  |  |  |  | 1 | 3 |  |
| H24 | 1 |  | |  |  |  |  |  | 1 |  |  |
| H25 | 5 |  | |  |  |  |  |  |  |  | 5 |
| H26 | 3 |  | |  |  |  |  |  |  |  | 3 |
| H27 | 1 |  | |  |  |  |  |  |  |  | 1 |
| H28 | 1 |  | |  |  |  |  |  |  |  | 1 |

Table S3. The eight microsatellite loci used in this study and the diversity estimates obtained from eight *Rhodeus notatus* populations on the Korean Peninsula

| Name | *A* | *A*_R_ | *H*_O_ | *H*_E_ | *F*_IS_ | *F*_ST_ | *R*_ST_ | p*R*_ST_ (95% CI) |
| --- | --- | --- | --- | --- | --- | --- | --- | --- |
| *Ak474*^63^ | 7 | 3.599 | 0.431 | 0.402 | -0.063 | 0.283 | 0.357 | 0.262 (0.125 – 0.398) |
| *Ak462*^63^ | 25 | 11.645 | 0.627 | 0.732 | 0.156 | 0.236 | 0.480 | 0.223 (0.049 – 0.457) |
| *RC600*^26^ | 12 | 5.257 | 0.182 | 0.174 | -0.049 | 0.785 | 0.980 | 0.687 (0.213 – 0.952) |
| *RC625*^26^ | 2 | 2.000 | 0.231 | 0.278 | 0.179 | 0.451 | 0.451 | 0.451 (0.451 – 0.451) |
| *RC772*^26^ | 8 | 4.330 | 0.306 | 0.370 | 0.143 | 0.166 | 0.333 | 0.160 (0.015 – 0.324) |
| *Ak424*^63^ | 22 | 9.303 | 0.384 | 0.555 | 0.308^*^ | 0.371 | 0.858 | 0.369 (0.117 – 0.665) |
| *Rser10*^62^ | 15 | 5.398 | 0.428 | 0.460 | 0.044 | 0.305 | 0.101 | 0.303 (0.099 – 0.580) |
| *Ak468*^63^ | 13 | 6.630 | 0.512 | 0.557 | 0.078 | 0.291 | 0.948 | 0.310 (0.039 – 0.770) |

Data include total number of alleles (*A*), allelic richness (*A*_R_), observed (*H*_O_) and expected (*H*_E_) heterozygosities, fixation indices (*F*_IS_), global *F*_ST_, *R*_ST_ and p*R*_ST_ (randomized *R*_ST_ using SPAGeDi).

^*^ *P* < 0.00625.

The number next to each locus is the reference number (in the main text) of the study where the primer set was first reported.

Table S4. Summary of six mitochondrial loci used to analyze the genetic diversity within and among populations of *Rhodeus notatus* on the Korean Peninsula.

| Locus | Primer | Sequence | *T*_a_ (°C) | Reference # |
| --- | --- | --- | --- | --- |
| COI | CO1e | CCAGAGATTAGAGGGAATCAGTG | 56 | 37 |
|  | CO1f | CCTGCAGGAGGAGGAGAYCC |  | 37 |
| NADH1 | GOBYL4201 | GTTGCMCAAACMATTTCHTATGAAG | 56 | 40 |
|  | GOBYH4937 | GGGGTATGGGCCCGAAAGC |  | 40 |
| NADH2 | GOBYL5464 | GGTTGAGGRGGCCTMAACCARAC | 54 | 40 |
|  | GOBYH6064 | CTCCTACTTAGAGCTTTGAAGGC |  | 40 |
| 16*S r*RNA | 16SF (16SA)^*^ | CGCCTGTTTAHCAAAAACAT | 56 | 38 |
|  | 16SR (16SB)^*^ | CCGGTYTGAACTCARATCA |  | 38 |
| 12*S r*RNA | 12S 19F | AAGCATAACACTGAAGATGTTAAG | 54 | Present study |
|  | 12S 1063B | CTCGGTGTAAGGGAGATG |  | Present study |
| Cyt *b* | LCB1 (L15267) | AATGACTTGAAGAACCACCGT | 54 | 39 |
|  | H16460 | CGAYCTTCGGATTACAAGACCG |  | 41 |

^*^ The primer sequence was slightly modified in this study.

Table S5. The species and sequence (NCBI GenBank accn #) information used for the BEAST tree analysis with divergence time shown in Figure S2 and Figure S3.

| Accession No. | Species name | Locality | Accession No. | Species name | Locality |
| --- | --- | --- | --- | --- | --- |
| AB366493 | *Rhodeus notatus* | South Korea | EF483937 | *Rhodeus uyekii* | South Korea |
| AB366491 | *Rhodeus notatus* | South Korea | AB366521 | *Rhodeus sinensis* | South Korea |
| AB366492 | *Rhodeus notatus* | South Korea | DQ396627 | *Rhodeus amurensis* | Russia |
| AB366488 | *Rhodeus notatus* | South Korea | AB366522 | *Rhodeus sinensis* | China |
| AB366489 | *Rhodeus notatus* | South Korea | AB366519 | *Rhodeus amarus* | Poland |
| KF410767 | *Rhodeus notatus* | South Korea | DQ396678 | *Rhodeus colchicus* | Georgia |
| KF410771 | *Rhodeus notatus* | South Korea | DQ396630 | *Rhodeus amarus* | Europe |
| KF410769 | *Rhodeus notatus* | South Korea | DQ396679 | *Rhodeus meridionalis* | Greece |
| KF410772 | *Rhodeus notatus* | South Korea | DQ396682 | *Rhodeus meridionalis* | Greece |
| KF410768 | *Rhodeus notatus* | South Korea | AB366518 | *Rhodeus sericeus* | China |
| KF410770 | *Rhodeus notatus* | South Korea | DQ396683 | *Rhodeus sericeus* | Russia |
| AB366487 | *Rhodeus notatus* | South Korea | AB366517 | *Rhodeus pseudosericeus* | South Korea |
| AB366486 | *Rhodeus notatus* | China | KF425517 | *Rhodeus pseudosericeus* | South Korea |
| AB366502 | *Rhodeus notatus* | China | AB366510 | *Rhodeus ocellatus ocellatus* | Taiwan |
| AB366490 | *Rhodeus notatus* | China | AB366514 | *Rhodeus ocellatus ocellatus* | South Korea |
| AB366503 | *Rhodeus notatus* | China | AB366512 | *Rhodeus ocellatus ocellatus* | China |
| AB366499 | *Rhodeus fangi* | China | AB366504 | *Rhodeus ocellatus kurumeus* | Japan |
| AB366497 | *Rhodeus fangi* | China | NC008642 | *Rhodeus ocellatus kurumeus* | Japan |
| AB366500 | *Rhodeus fangi* | China | AB366508 | *Rhodeus ocellatus ocellatus* | China |
| AB366501 | *Rhodeus fangi* | China | HQ113265 | *Rhodeus ocellatus* | China |
| KF410764 | *Rhodeus fangi* | China | AB366515 | *Rhodeus ocellatus ocellatus* | China |
| AB366498 | *Rhodeus fangi* | China | AB366511 | *Rhodeus ocellatus ocellatus* | China |
| AB366495 | *Rhodeus atremius suigensis* | Japan | AB366516 | *Rhodeus ocellatus ocellatus* | China |
| KF410761 | *Rhodeus atremius suigensis* | Japan | FJ515921 | *Acheilognathus somjinensis* | South Korea |
| KF410760 | *Rhodeus atremius suigensis* | Japan | NC013704 | *Acheilognathus koreensis* | South Korea |
| AB366494 | *Rhodeus atremius suigensis* | Japan | EF483933 | *Acheilognathus intermedia* | South Korea |
| AB366484 | *Rhodeus atremius atremius* | Japan | DQ396628 | *Acheilognathus chankaensis* | Russia |
| AB366485 | *Rhodeus atremius atremius* | Japan | NC008668 | *Acheilognathus typus* | Japan |
| AB366496 | *Rhodeus atremius suigensis* | Japan | NC013711 | *Acheilognathus macropterus* | South Korea |
| KF410759 | *Rhodeus atremius atremius* | Japan | AB620135 | *Acheilognathus rhombeus* | Japan |
| AB366527 | *Rhodeus rheinhardti* | Vietnam | AB620134 | *Acheilognathus cyanostigma* | Japan |
| AB369282 | *Rhodeus laoensis* | Laos | AB620146 | *Acheilognathus tabira nakamurae* | Japan |
| AB366524 | *Rhodeus spinalis* | China | AB620136 | *Acheilognathus melanogaster* | Japan |
| AB366523 | *Rhodeus spinalis* | China | EU241475 | *Tanichthys albonubes* | Aquarium |
| AB366520 | *Rhodeus sinensis* | China | KC524521 | *Kichulchoia brevifasciata* | South Korea |


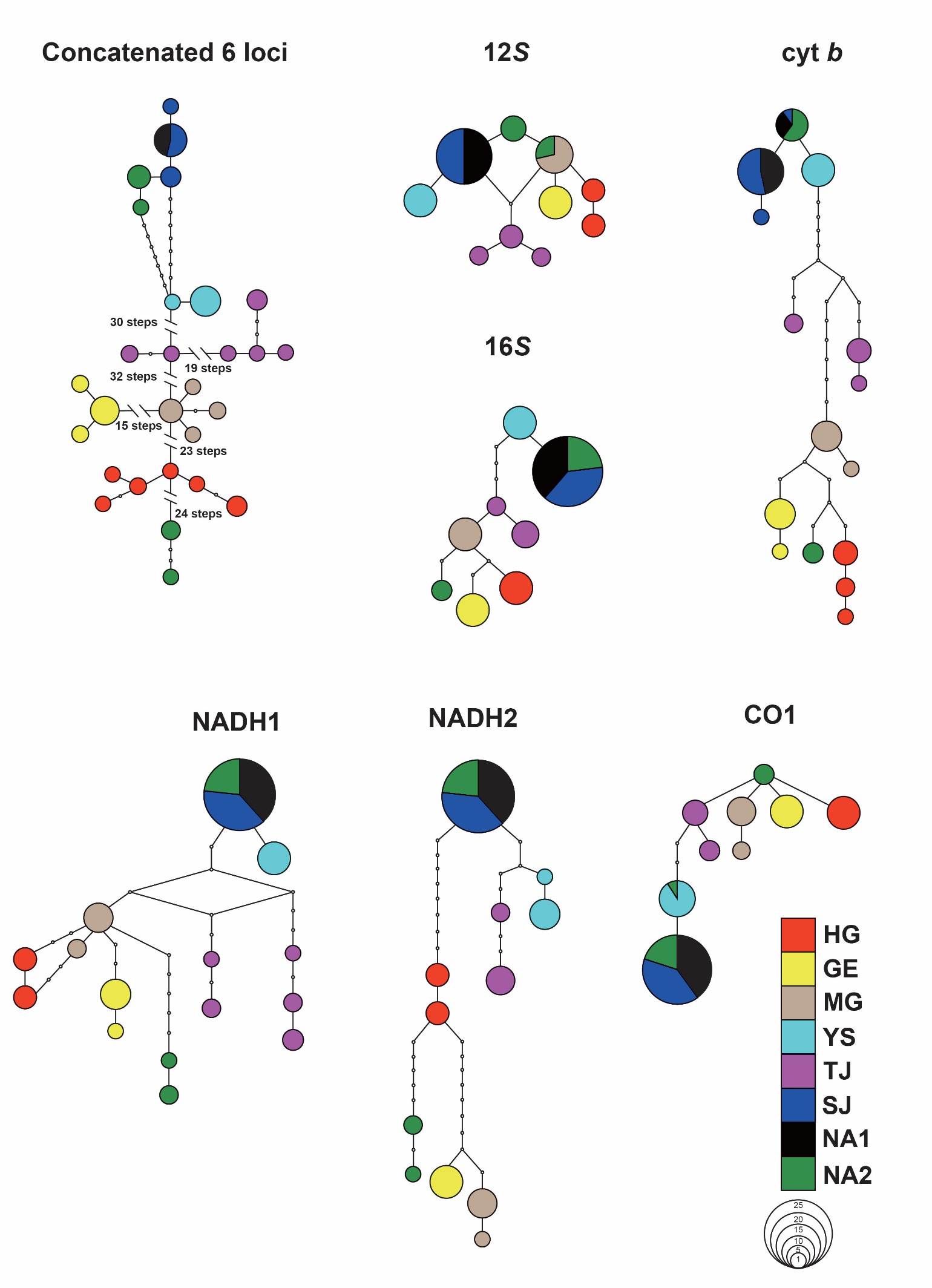


Figure S1. The unrooted haplotype network generated based on six mitochondrial loci used.


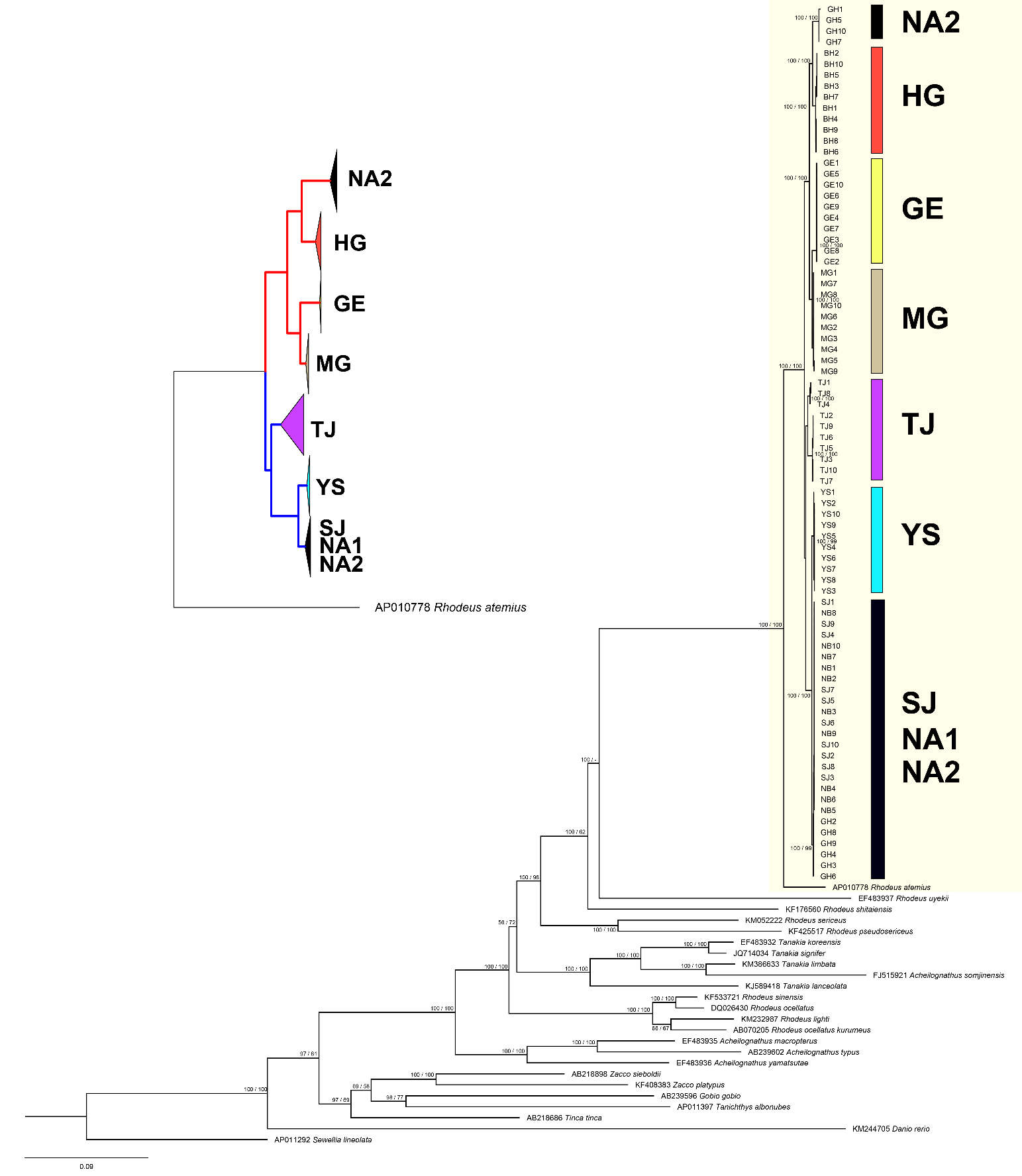


Figure S2. Phylogenetic tree of *R. notatus* and the related species (Acheilognathidae and Cyprinidae; see supplementary Table S5) reconstructed using 6 mitochondrial loci used in this study based on Bayesian inference (BI) and maximum likelihood (ML) algorithms. BI posterior probabilities (in percent) and ML bootstrapping values were put on the nodes.


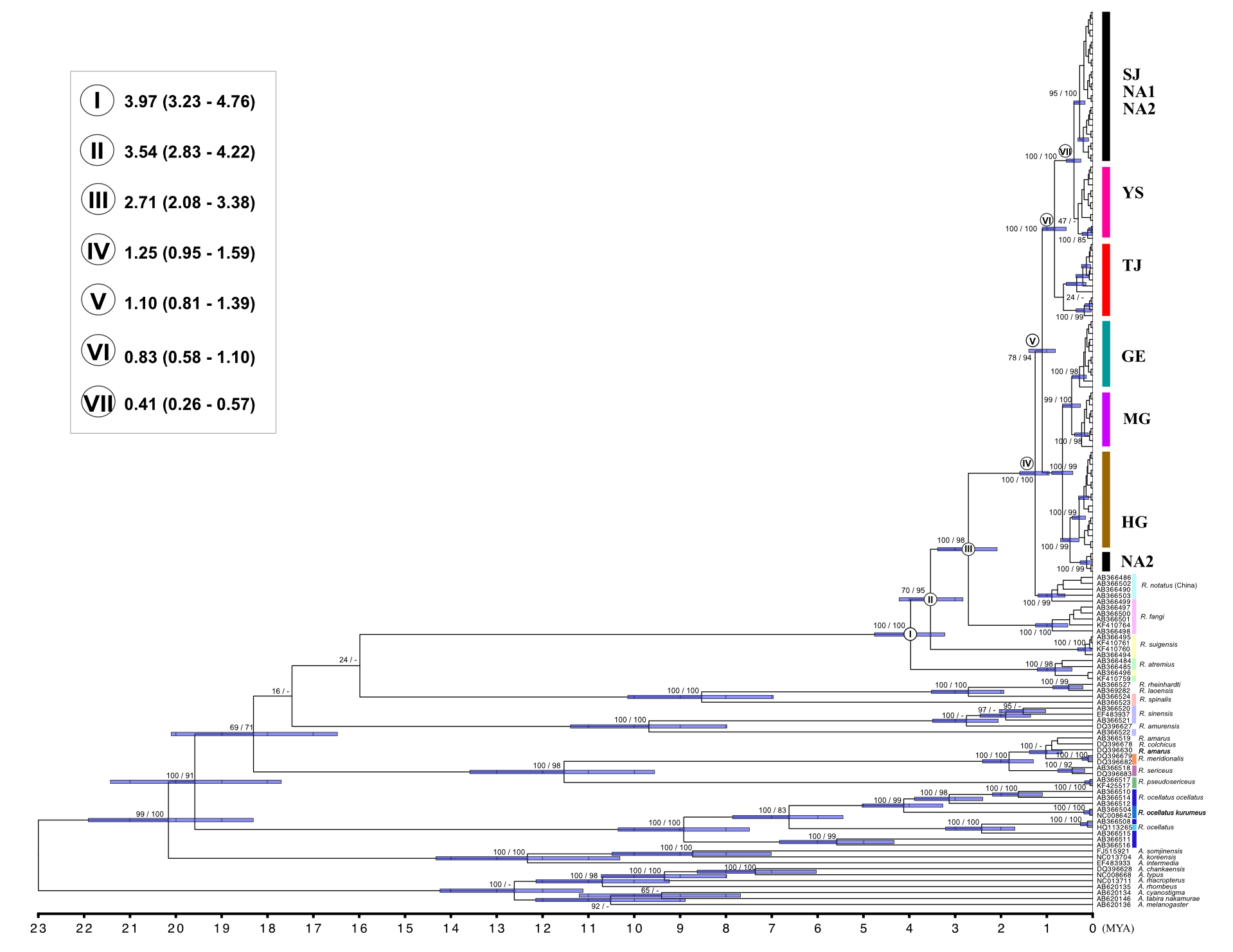


Figure S3. Time-calibrated Bayesian tree reconstructed by BEAST 2.3.2 using cyt *b* sequences of *Rhodeus notatus* and various Acheilognathid species (see supplementary Table S5). BI posterior probabilities (in percent) and ML bootstrapping values were put on the nodes.


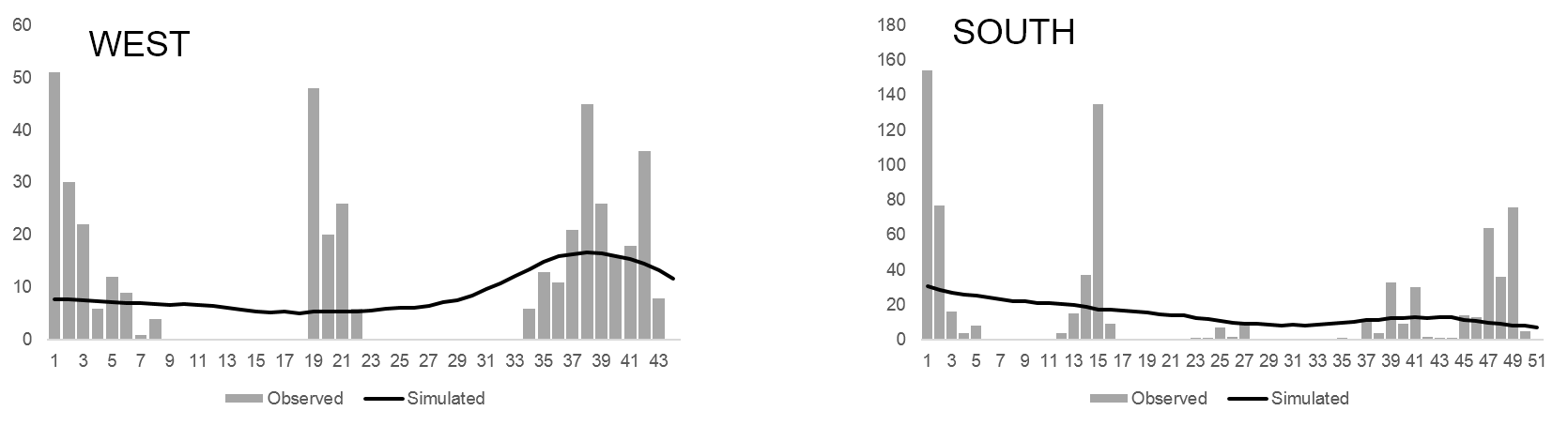


Figure S4. The results of mismatch distribution analysis performed using six mitochondrial loci for western (a) and southern population groups (b) of *Rhodeus notatus* on the Korean Peninsula.


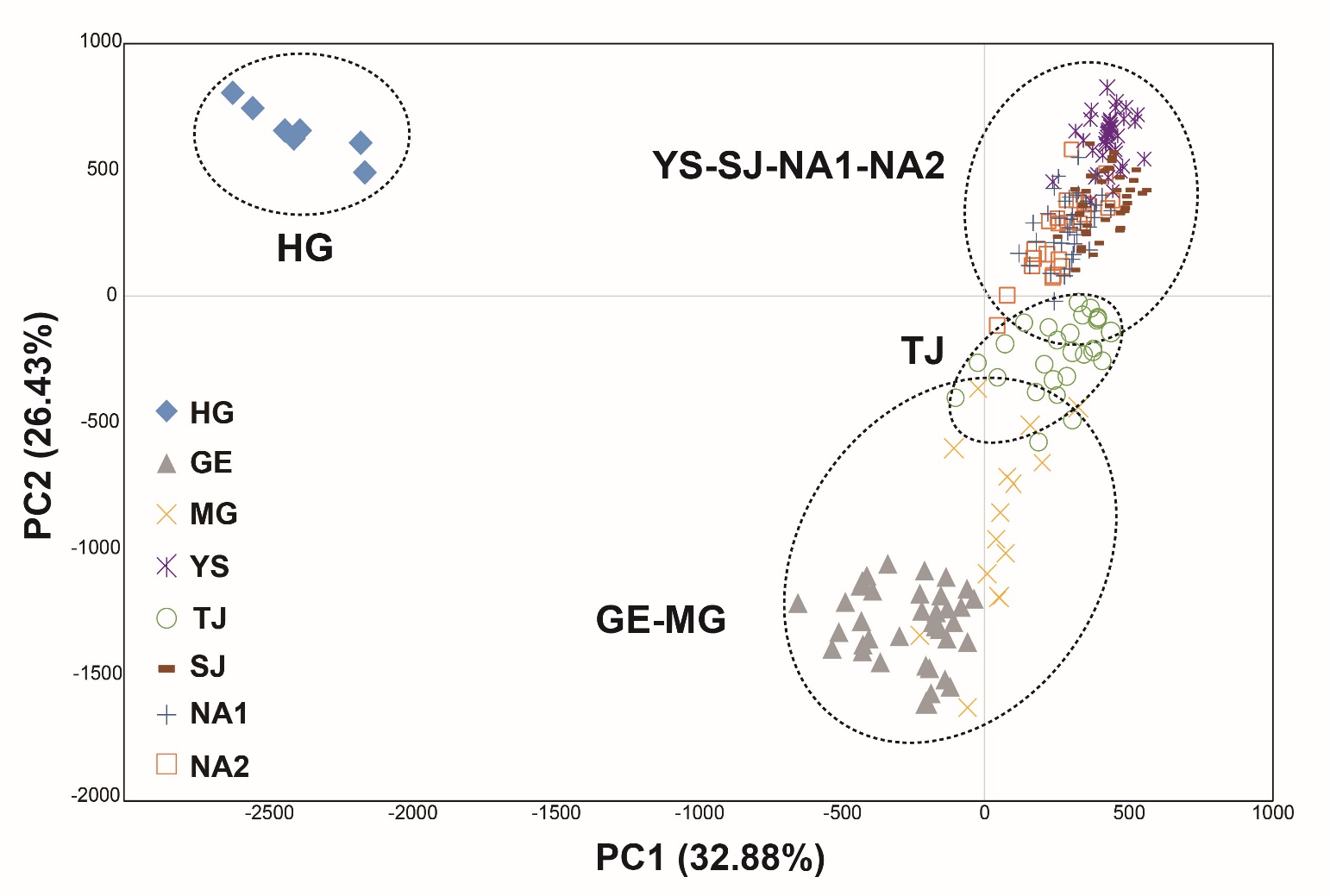


Figure S5. Bi-dimensional plot of the principal component analysis (PCA) showing genetic differentiation based on pairwise-FST values among eight populations of *Rhodeus notatus* from the Korean Peninsula. See Table 1 or Fig. 1 for locality codes.
